# HIV-1_LAI_ Nef blocks the development of hematopoietic stem/progenitor cells into myeloid-erythroid lineage cells

**DOI:** 10.1186/s13062-021-00317-3

**Published:** 2021-12-20

**Authors:** Wei Zou, Juanjuan Xing, Shijie Zou, Mei Jiang, Xinping Chen, Qi Chen, Daozheng Liu, Xiangcheng Zhang, Xin Fu

**Affiliations:** 1 Department of Infectious Diseases, The 1St Affiliated Hospital of Nanchang University, Nanchang, 330006 Jiangxi China; 2 Department of Burn, The 1st Affiliated Hospital of Nanchang University, Nanchang, 330006 Jiangxi China; 3 Department of Experimental Medicine, The 1st Affiliated Hospital of Nanchang University, Nanchang, 330006 Jiangxi China; 4 Department of Gynecology and Obstetrics, The 1st Affiliated Hospital of Nanchang University, Nanchang, 330006 Jiangxi China; 5 Jiangxi Provincial Key Laboratory of Preventive Medicine, School of Public Health, Nanchang University, Nanchang, 330006 Jiangxi China

**Keywords:** LAI Nef, Hematopoietic stem/progenitor cells, Myeloid-erythroid lineage cells, Humanized mouse model, Differentiation

## Abstract

**Background:**

A variety of hematopoietic abnormalities are commonly seen in human immunodeficiency virus-1 (HIV-1) infected individuals despite antiviral therapy, but the underlying mechanism remains elusive. Nef plays an important role in HIV-1 induced T cell loss and disease progression, but it is not known whether Nef participates in other hematopoietic abnormalities associated with infection.

**Results:**

In the current study we investigated the influence of HIV-1_LAI_ Nef (LAI Nef) on the development of hematopoietic stem/progenitor cells (HSPCs) into myeloid-erythroid lineage cells, and found that *nef* expression in HSPCs blocked their differentiation both in vitro and in humanized mice reconstituted with *nef*-expressing HSPCs.

**Conclusions:**

Our novel findings demonstrate LAI Nef compromised the development of myeloid-erythroid lineage cells, and therapeutics targeting Nef would be promising in correcting HIV-1 associated hematopoietic abnormalities.

**Supplementary Information:**

The online version contains supplementary material available at 10.1186/s13062-021-00317-3.

## Background

Human immunodeficiency virus type 1 (HIV-1) is the etiological cause of acquired immunodeficiency syndrome (AIDS). The major targets for HIV-1 infection are CD4 + T cells and macrophages but HIV-1 can also infect hematopoietic stem/progenitor cells (HSPCs) and establish latency in vitro and in vivo*.* HSPC infection may cause abnormal hematopoiesis in HIV-1 infected individuals [1–5]. Further, CD34 + HSPCs isolated from either the peripheral blood of infected individuals or from the cord blood sampled following births of HIV-1 negative babies born to HIV-1 positive mothers, showed reduced growth/differentiation in vitro [6, 7]. This suggests compromised functionality of CD34 + HSPCs in the presence of HIV-1. In addition, the severity of hematopoietic abnormalities was found to be positively correlated with HIV-1 disease progression with pancytopenia often occurring at the end stage of the disease [8].

Although antiretroviral therapy (ART) partially alleviates the suppression of HIV-1 on the hematopoietic system, the normal maturation of hematopoietic cells is not completely restored [2]. As shown by Sauce and her colleagues, persistent damage to the lymphopoietic system or exhaustion of lymphopoiesis was observed in HIV-1 infected individuals despite suppression of replication by ART. Persistent damage to lymphopoiesis was also observed in elite controllers [9]. Besides the cytotoxic effect due to direct HIV-1 infection of HSPCs as indicated by some studies, these observations may also suggest that factors besides viral replication participate in the suppression of hematopoeisis by HIV-1[1, 3, 4]. Viral-encoded proteins are important candidates to consider in this process.

As one of the early transcriptional products in HIV-1 life cycle and one of the viral proteins that are packaged in the viral particles, Nef plays an important role in HIV-1 pathogenesis and disease progression. In addition to its intracellular activities, Nef is secreted from cells [10–14]. Nef’s concentration in the serum of HIV-1 infected individuals is about 1–10 ng/mL[15]. It is also found that extracellular Nef can accumulate inside uninfected cells through endocytosis, extracellular vesicles or exosomes and other mechanisms. Besides, Nef can be transferred between infected and uninfected cells through cell–cell contact, and from *nef*-expressing cells to bystander cells through intercellular nanotubular conduits [16–18]. In these circumstances Nef protein may serve as a form of intercellular communication. These findings suggest that Nef could be present and execute its functions in HSPCs as the result of direct infection of stem/progenitor cells by HIV-1 and/or by entry of extracellular Nef into these cells via above mentioned mechanisms. However, whether or not Nef participates in the suppressive effect of HIV-1 on HSPC still remains elusive. In this study we investigated the effect of Nef on the development and differentiation of HSPCs into myeloid-erythroid lineage cells in vitro and in vivo. We found that intracellular expression of Nef in HSPCs blocked the development and production of myeloid-erythroid lineage cells as demonstrated by reduced formation of myeloid and erythroid colonies in vitro*.* In humanized mice there were greatly reduced numbers of mature monocytes/macrophages and erythroid cells.

## Results

### *Development of nef-expressing HSPCs into myeloid and erythroid lineage cells was compromised *in vitro

**Fig. 1:**
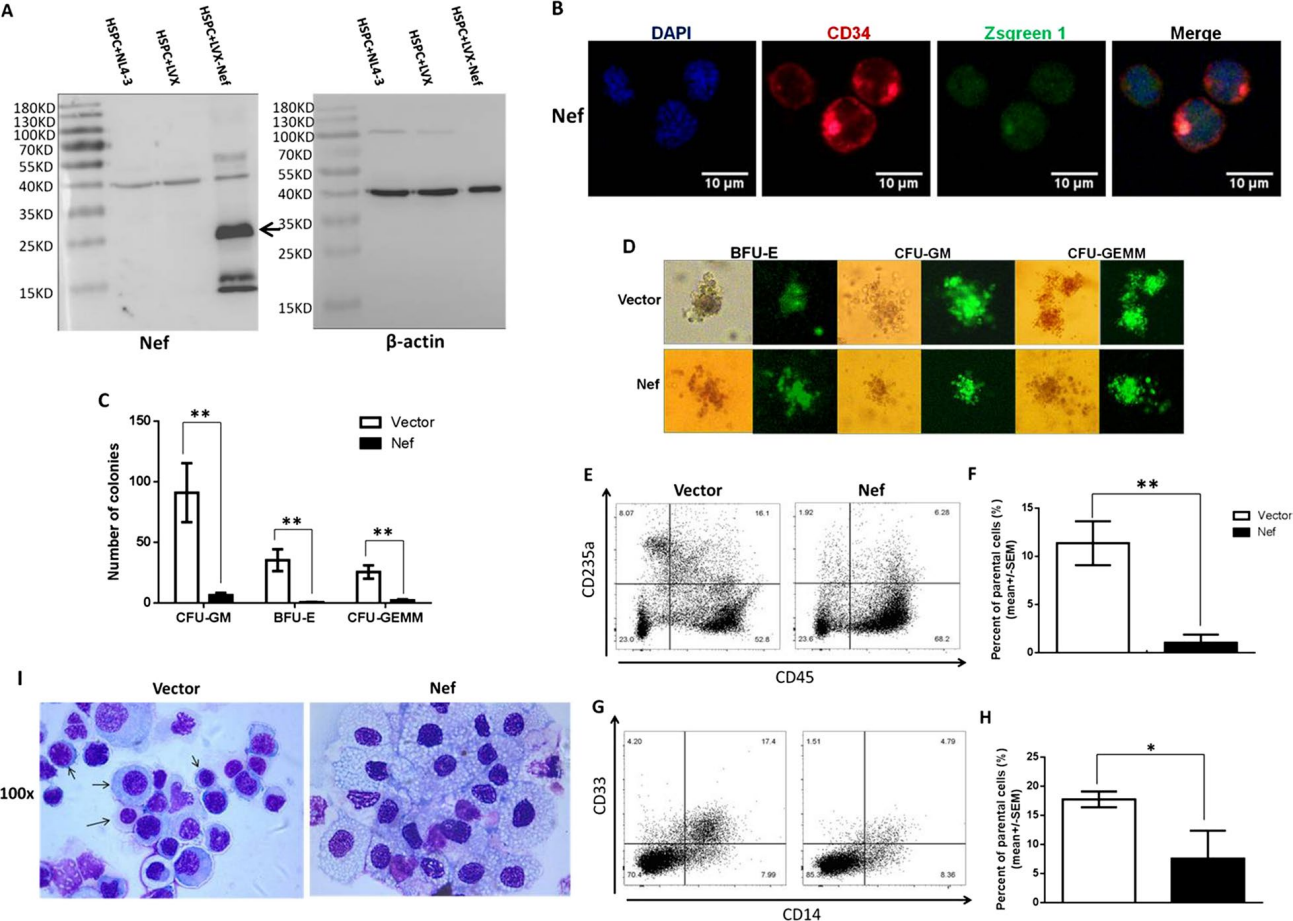
Effects of Nef on erythroid and myeloid cell development in vitro. Expression of Nef protein in CD34 + cells either transduced with LVX-vector or LVX-nef, or infected by NL43 was confirmed by Western blotting with a Nef specific antibody. Beta-actin was used as a loading control. The results are shown in **a**. CD34 and Nef (zsgreen1) expression and localization in the CD34 + cells transduced with LVX-nef was examined with immunofluorence staining and the results are shown in **b**. LVX-vector or nef transduced HSPCs were inoculated into methylcellulose-based media for colony forming assay. After 14 days in culture, numbers of CFU-GM, BFU-E and CFU-GEMM colonies were scored. Data are represented as mean ± SEM in **c**. At least 7 independent experiments were performed. Colonies formed in the methylcellulose-based media was shown in **d**. These colonies were harvested and some of the harvested cells were stained for CD45/CD235a (erythroid, **e** and **f**) and CD45/CD33/CD14 (myeloid, G and H) markers for flow analysis. Representative flow images of erythroid and myeloid lineage cells were shown in **e** and **g**, and the cumulative flow data of these two lineage cells were shown in **f** and **h**. The parental cells in **f** and **h** are referred to live cells gated in FSC/SSC plot and CD45 + cells, respectively. The rest of the harvested cells were subjected to cyto-spinning and Giemsa staining, and representative images of these cells are shown in **i**. Small arrows in **i** refer to erythroid cells at different developing stages. Data are represented as mean ± SEM. At least 3 independent experiments were performed. **p < 0.01; *p < 0.05

In order to study the effect of Nef on the development of HSPCs into myeloid and erythroid lineage cells and in consideration of the low efficiency of direct infection of HSPCs with HIV-1 in vitro, in current study we delivered *nef* via lentiviral (LVX-EF1α-LAINef-IRES-zsgreen1, LVX-nef) transduction into CD34 + HSPCs enriched from human cord blood. The ZsGreen1 marker is necessary to sort the transduced cells prior to downstream in vitro and in vivo experiments. Expression of Nef protein was confirmed in flow cytometry-enriched CD34 + Zsgreen1 + cells in our previously published data [19] and in LVX-nef transduced HSPCs (Fig. 1a) by Western blotting with a Nef specific antibody. Nef expression in CD34 + cells was also demonstrated by co-localization of CD34 + staining and zsgreen1 in a single cell (Fig. 1b). Development of *nef*-expressing CD34 + cells into myeloid and erythroid lineage cells was examined using the colony forming assay (CFA). We chose LAI Nef for study because it has been shown that X4-tropic viruses are likely to be present when myeloid and erythroid hematopoietic abnormalities appear and because X4-tropic viruses infect a broader range of HSPCs than R5-tropic viruses [1], which may account in part for accelerated disease progression when X4-tropic viruses are present. Also, LAI exhibits massive, Nef-dependent CD4 + T cell killing in bone/liver/thymus (BLT) humanized mice [20, 21].

After 14 days in culture colonies formed in the MethoCult™ media of CFA assay were scored and it was found that the numbers of erythroid colonies (BFU-E), myeloid colonies (CFU-GM), and the colonies containing both erythroid and myeloid lineage cells (CFU-GEMM) were drastically reduced by *nef* expression in HSPCs (Fig. 1c). Zsgreen1 expression in the colonies was shown in Fig. 1d, and *nef* expression in these colonies was confirmed by RT-qPCR with *nef* specific primers and probe (Additional file 1: Fig. S1). To further quantitate the effect of Nef on myeloid and erythroid cell development, cells harvested from the CFA plates were stained for erythroid and myeloid cell markers and examined using flow analysis. Our results showed that *nef* expression in HSPCs led to a significant decrease in the percentage of CD45-CD235a + erythroid cells (Fig. 1e and f) suggesting Nef may be involved in the developmental abnormality of erythroid lineage cells and subsequent anemia in HIV-1 infected individuals. For myeloid lineage cells we observed a significant reduction of the percentage of CD45 + CD14 + CD33 + cells developed from *nef-*expressing HSPCs (Fig. 1g and h). CD14 is a specific monocyte/macrophage marker, and CD33 is expressed mainly on non-terminally differentiated immature myeloid cells [22]. It is reported that CD33 expression is a feature of multi-potent hematopoietic stem cells, but not of true stem cells, and its levels decrease with maturation and differentiation, indicating its potential role in the regulation of myeloid cell differentiation [23]. Upon maturation CD33 expression is lost. Therefore, our results of decreased CD14 + CD33 + cells suggest that Nef disturbed myeloid cell maturation. Consistent with our results, some studies reported that HIV-1 Nef not only negatively regulated cell surface expression of macrophage colony-stimulating factor (M-CSF) receptor but also interfered with M-CSF receptor signaling thus inhibiting M-CSF bioactivities and causing monocyte/macrophage differentiation deficiency [24, 25].

To find out whether *nef* expression induces morphological change of developing HSPCs, colonies from the CFA plates were transferred to a slide and stained with Giemsa staining. Consistent with the flow data, majority of cells on the slide were found to be myeloid cells and as shown in Fig. 1i erythroid lineage cells were hardly seen in the presence of Nef while these cells at different developing stages can be seen in the control group (small arrows). Besides, in the presence of Nef enlarged foamy cells were more often observed and nuclear debris and karyopyknosis was occasionally seen indicating apoptosis (Fig. 1i). As reported in our previous work [19], more LVX-nef transduced CD34 + cells were dead than LVX-vector transduced cells supporting the finding that LAI Nef hindered the development and production of myeloid and erythroid lineage cells at least by causing their premature death.

**Fig.2:**
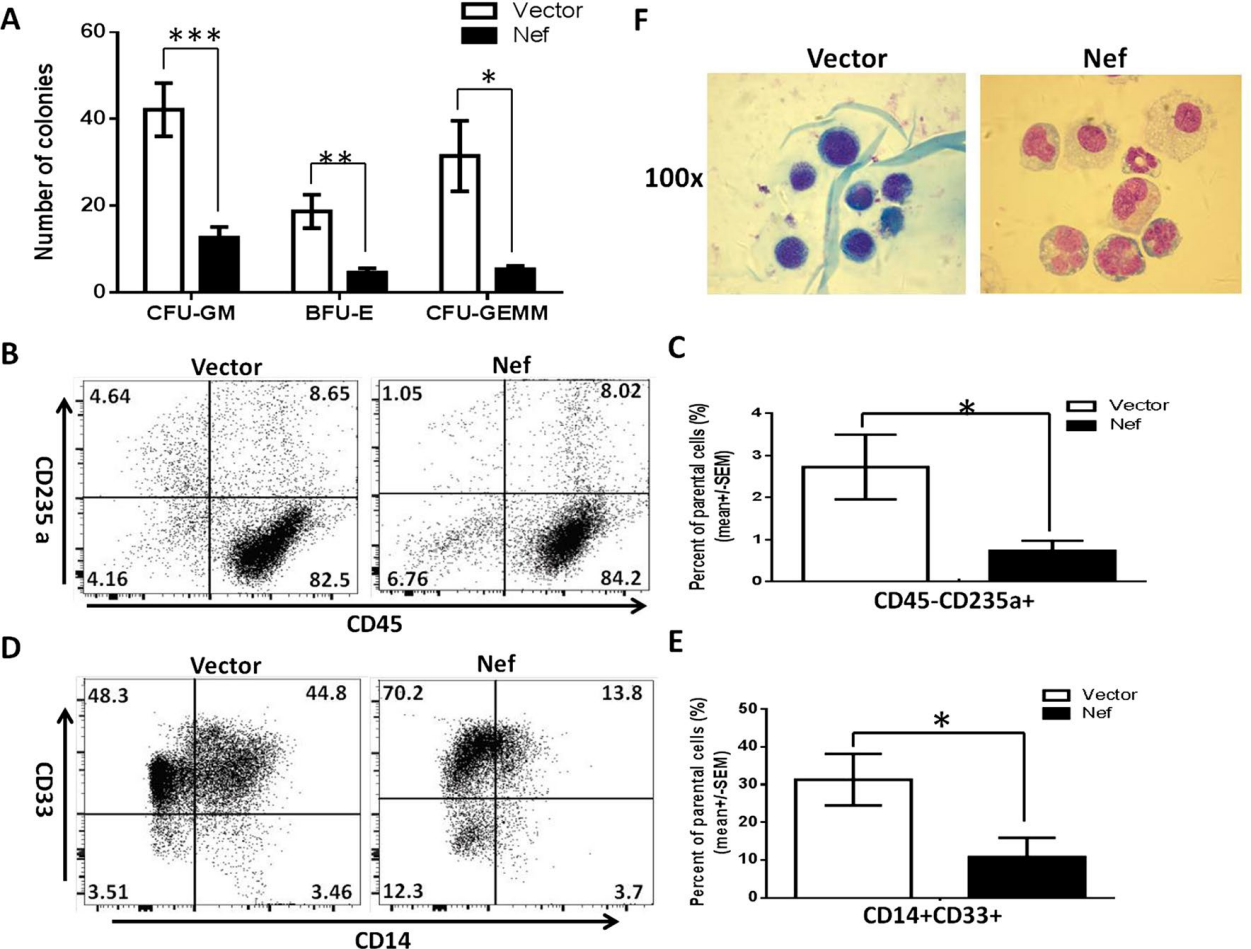
Effects of the Nef from a primary isolate on erythroid and myeloid cell development in vitro. LVX-vector or nef transduced HSPCs were inoculated into methylcellulose-based media for colony forming assay. After 14 days in culture, numbers of CFU-GM, BFU-E and CFU-GEMM colonies were scored. Data are represented as mean ± SEM in **a**. These colonies were harvested and some of the harvested cells were stained for CD45/CD235a (erythroid, **b** and **c**) and CD45/CD33/CD14 (myeloid, **d** and **e**) markers for flow analysis. Representative flow images of erythroid and myeloid lineage cells were shown in **b** and **d**, and the cumulative flow data of these two lineage cells were shown in **c** and **e**. Data are represented as mean ± SEM. The parental cells in **c** and **e** are referred to live cells gated in FSC/SSC plot and CD45 + cells, respectively. The rest of the harvested cells were subjected to cyto-spinning and Giemsa staining, and representative images of these cells are shown in **f**. ***p < 0.001; **p < 0.01; *p < 0.05

Of the known functions of Nef LAI is fully functional. To find out whether similar results from LAI Nef can be observed for a Nef from a primary isolate, we chose a primary nef that was the least identical (Additional file 1: Fig S2, percent identity: 80.65%) in sequence with LAI Nef in our hands and tested its effects on the development of myeloid and erythroid lineage cells in vitro with the same methods. As shown in Fig. 2, the numbers of BFU-E, CFU-GM and CFU-GEMM colonies were significantly reduced by primary *nef* expression in HSPCs (Fig. 2a). To further quantitate the effect of Nef on myeloid and erythroid cell development, cells harvested from the CFA plates were stained for erythroid and myeloid cell markers and examined using flow analysis. Consistent with the results from LAI Nef, primary *nef* expression in HSPCs led to a significant decrease in the percentage of CD45-CD235a + erythroid cells (Fig. 2b and c) and CD45 + CD14 + CD33 + myeloid cells (Fig. 2d and e). Giemsa staining of the colonies indicated that the majority of cells on the slide were myeloid cells and as shown in Fig. 2f erythroid lineage cells were very few in the presence of Nef when compared with the control group. Besides, Nef expression caused more enlarged foamy and apoptotic cells (Fig. 2f). These results demonstrated that the Nef from a primary isolate that shared only 80.65% of homology in sequence with LAI Nef had similar inhibitory effects on hematopoiesis as LAI Nef.

### Development of nef-expressing HSPCs into myeloid and erythroid lineage cells was compromised in humanized mice

**Fig.3:**
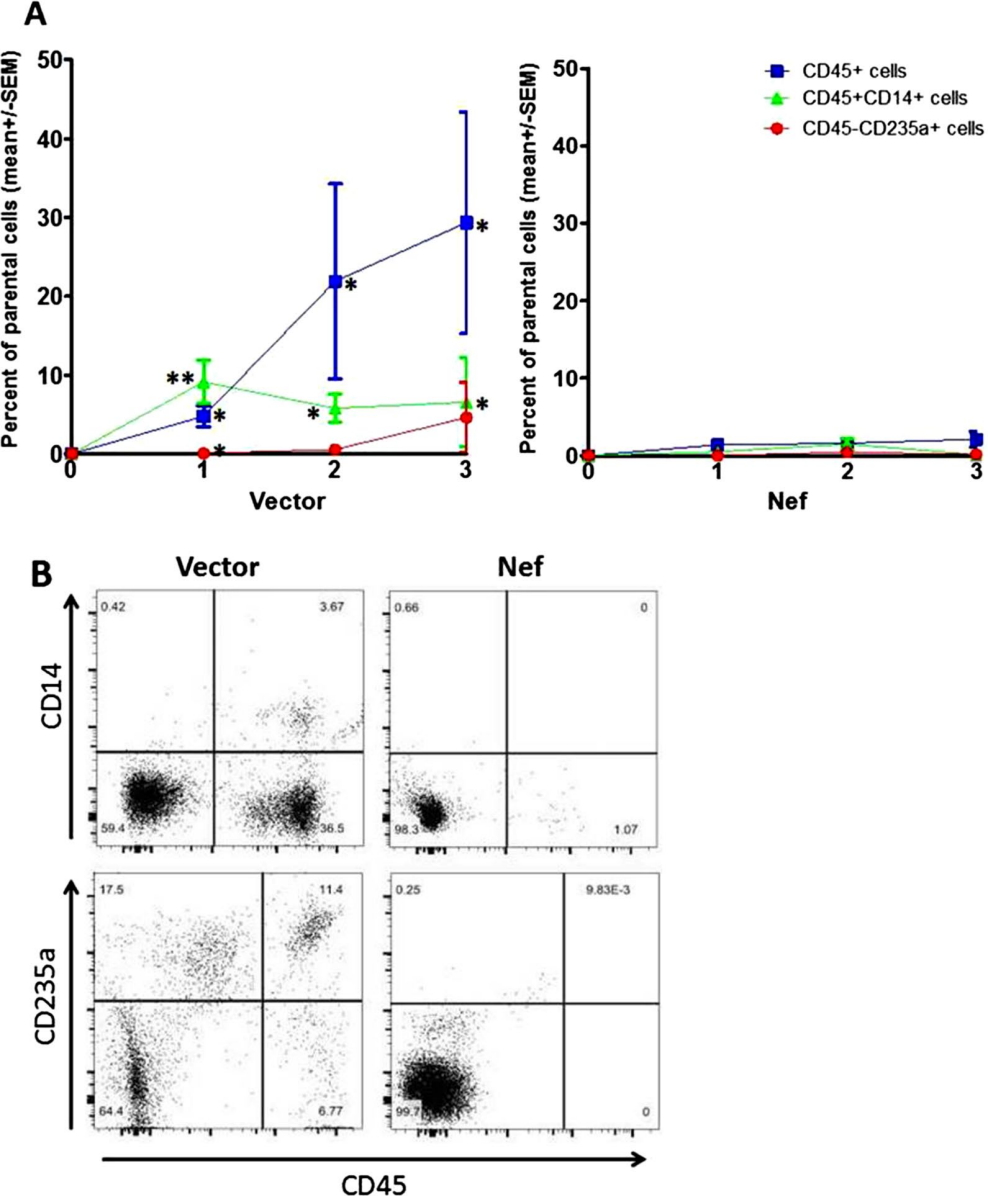
Longitudinal effects of Nef on CD14 + myeloid and CD235a + erythroid cells in the peripheral blood of humanized mice. LVX-vector or LVX-nef transduced CD34 + cells were transplanted into irradiated NCG mice. Every month (for 3 months) after transplantation, mice were bled and peripheral blood was stained for CD45/CD235a and CD45/CD14 for flow analysis. The percentages of CD45 + CD14 + and CD45-CD235a + cells were compared between vector and nef group referring to myeloid and erythroid cells, respectively. Representative flow images of the blood cells are shown in **b**, and the cumulative flow data of three bleedings of all the mice in each group are shown in **a** (n = 6 for vector group and n = 7 for nef group). The gating strategy was live > CD45-CD235a + for erythroid and live > Zsgreen +  > CD45 + CD14 + for myeloid cells, respectively. **p < 0.01; *p < 0.05

To study the effect of Nef on the development of HSPCs into myeloid and erythroid lineage cells in vivo, we transplanted irradiated NCG mice with the HSPCs transduced with either LVX-vectoror the LVX expressing *nef* (LVX-nef). Development of human myeloid and erythroid cells in the peripheral blood of these humanized mice was monitored every 4 weeks. Consistent with the in vitro results, *nef* expression greatly reduced the development of total CD45 + cells and CD45 + CD14 + (myeloid) cells in peripheral blood. For erythroid cells (CD45-CD235a +) at one month after transplantation *nef* expression resulted in slightly but significantly decreased levels of CD235a + erythroid cells (mean of control versus Nef group: 0.058 vs. 0.014, *p* < 0.05). But this difference disappeared with time and by the third month there was no difference in the levels of CD235a + erythroid cells between Nef group and control group (Fig. 3a). These results indicated that Nef expression eliminated both myeloid and erythroid cells from the peripheral blood of humanized mice. Representative flow images of the bleedings are shown in Fig. 3b.

**Fig.4:**
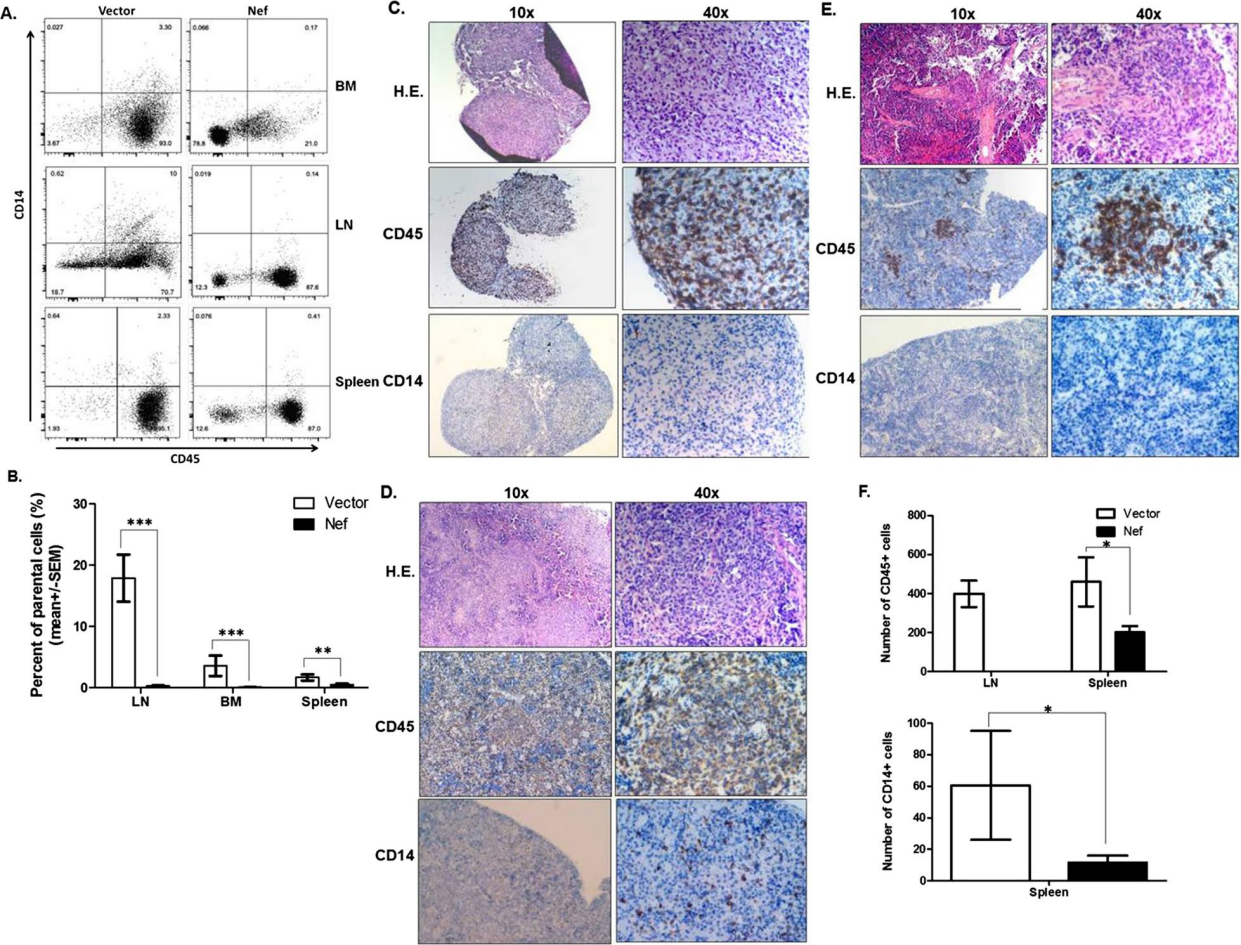
Effects of Nef on CD45 + CD14 + myeloid cells in the bone marrow, spleen and lymph nodes of humanized mice. LVX-vector or LVX-nef transduced CD34 + cells were transplanted into irradiated NCG mice. Three months after transplantation, mice were sacrificed and bone marrow, spleen and lymph nodes were harvested for CD45 + CD14 + cell analysis with flow cytometry. Representative flow images were shown in **a**, and the cumulative flow data of all the mice in each group were shown in **b**. The gating strategy was live > Zsgreen +  > CD45 + CD14 + . Spleens and lymph nodes were also paraffin embedded for H&E staining and IHC staining of CD45 + and CD14 + cells. Representative images of H&E and IHC staining from each group are shown in **c** (control group lymph node), **d** (control group spleen) and **e** (Nef group spleen), and the quantitative data of CD45 + and CD14 + cells of IHC staining in each group are shown in **f** (n = 6 for vector group and n = 7 for Nef group). *p < 0.05; **p < 0.01; ***p < 0.001

Three months after transplantation, mice were sacrificed and bone marrow, spleen, and lymph nodes were harvested for flow cytometry analysis. Spleens were further analyzed by H&E and immunohistochemistry staining for CD45 + CD14 + cells. Via flow analysis the percentages of CD45 + CD14 + cells in these three organs were found significantly decreased in Nef group when compared with the control group (Fig. 4a and b). H&E staining of the lymph nodes isolated from control mice showed somewhat a normal structure consisting of cortex and medulla. Consistent with flow data, via IHC staining, plenty of CD45 + cells and very few CD14 + cells were detected in the lymph nodes of control mice (Fig. 4c). However, lymph nodes could hardly be found in the humanized mice transplanted with *nef*-transduced HSPCs so that all the lymph nodes harvested from the Nef mice were only sufficient for flow analysis, indicating that Nef affected the development of hematopoietic cells and the formation of lymph nodes.

In the spleens of control mice white pulp and red pulp were easily recognized in H&E staining. In IHC staining CD45 + cells were found extensively reconstituted and CD14 + cells were sporadically seen in the spleen of control mice (Fig. 4d). However, in the mice transplanted with *nef*-transduced HSPCs, the regions of white pulp and red pulp in spleen were obscure in H&E staining. In IHC staining sporadic clusters of CD45 + cells and almost none of CD14 + cells were detected (Fig. 4e). Quantitative counting of CD45 + and CD14 + cells in the lymph nodes and spleens of control and Nef mice were conducted and the results showed that CD45 + cells in both the lymph nodes and spleens and CD14 + cells in the spleens of the Nef mice were significantly reduced in number compared with control mice (Fig. 4f). These results further indicated that Nef affected the development and reconstitution of CD45 + and CD14 + cells in the lymphoid organs of humanized mice. Nef expression was confirmed in the spleens of all the humanized mice transplanted with the HSPCs transduced with LVX-nef as demonstrated in our previous work [19]. These results indicate that Nef was clearly present in the humanized mice and executed its biological functions.

## Discussion

Potent ART is effective in suppressing but not eradicating HIV-1 replication. With more sensitive methods a number of studies reported detectable low-level viremia after ART suppressed viral replication to undetectable level by standard assays [26–31]. Through mathematic modeling it was suggested that a very small fraction of infected cells was capable of surviving indefinitely while continuing to produce virus. Furthermore, in many patients much of the residual plasma virus belongs to a single predominant clone suggesting that it again might arise from slowly dividing virus-producing cells. Thus, it was proposed that latently infected stem cells contributed to clonal viral expansion upon intermittent activation or viral blips observed in HIV-1 infected patients on successful antiviral therapy [26, 31, 32].

A variety of hematopoietic abnormalities including anemia and neutropenia are often observed in HIV-1infected individuals [33]. The findings that HIV-1 can infect both hematopoietic stem/progenitor cells (HSPC) [16, 21, 24, 34] and stromal cells in the bone marrow [35] raised the possibility that dysfunction of these cells due to HIV-1 infection adversely affects hematopoiesis. Supporting this hypothesis here we report a Nef-dependent loss of colony-forming cell growth and compromised proper hematopoietic lineage differentiation of HSPCs in vitro and in vivo. It has long been known that HIV-1 inhibited multi-lineage hematopoiesis of CD34 + cells isolated from HIV-1 infected Thy/liv implants of SCID-hu mice. In this model HIV-1 not only caused severe depletion of human thymocytes but also severely decreased the ex vivo recovery of human progenitor cells capable of differentiation into both erythroid and myeloid lineages [36]. Importantly, CD34 + cells were not lost during infection. This phenomenon was more prominent for X4-tropic viruses. More importantly, hematopoiesis ex vivo was not fully restored upon suppression of viral replication by ART in the Thy/liv mice. This finding suggests that as in HIV infected individuals there are factors independent of viral replication that contribute to hematopoietic abnormality observed [36]. Models other than the SCID/hu mouse model are limited in scope. The CD4C/HIV Tg mouse model only expresses Nef in CD4 expressing cells excluding erythroid development from analysis and the fetal thymic organ culture model is restricted to thymic development. Nor have any of these models been capable of addressing the question of which viral factor(s) are responsible for defects in erythroid development. Here we have demonstrated that the effect of HIV-1 infection on erythroid development suggested by the results with the SCID/hu mouse experiments that can be reproduced by expression of *nef* in CD34 + cells.

Previous studies found that HIV-1 envelop glycoprotein gp120 and TNF-α produced by virus or envelop glycoprotein-stimulated blood monocytes and bone marrow mononuclear cells mediated HIV-1 induced inhibition of hematopoiesis [37, 38]. HIV-1 p24 gag protein was also shown to inhibit the formation of myeloid colonies of bone marrow cultures but to have a minor effect on the formation of erythroid colonies [39]. As a follow-up of our previous findings that LAI Nef is the dominant viral factor responsible for thymocyte depletion in BLT humanized mice, which was independent of viral replication [21], we investigated whether LAI Nef affected the development of HSPCs into myeloid and erythroid lineage cells. We found that LAI Nef inhibited the colony forming ability of CD34 + HSPCs into these two lineage cells in vitro as demonstrated by reduced numbers of CFU-GM, BFU-E and CFU-GEMM colonies in CFA assay. These results were further confirmed via FACS analysis of CD235a + erythroid and CD33 + /CD14 + myeloid cells of above colonies. Giemsa staining of the colonies revealed that *nef* expression in HSPCs caused reduced number of erythroid and myeloid cells. In addition, *nef* expression in HSPCs induced enlarged foamy myeloid cells and nuclear debris and karyopyknosis in myeloid cells. These results indicate that Nef compromised the colony forming ability and differentiation of CD34 + HSPCs in vitro by causing them premature death. Similar results were obtained with the Nef from a primary isolate that was 80.65% identical in sequence to LAI Nef, indicating that the inhibitory effect of Nef on hematopoiesis probably is a property of most Nefs that are from either lab-adapted HIV strains or primary isolates. To further confirm the effect of Nef on myeloid and erythroid cell development observed in vitro, *nef*-transduced HSPCs were transplanted into immune deficient mice and were monitored for development into these 2 lineage cells periodically in peripheral blood and tissues. Consistent with in vitro findings, Nef expression in HSPCs blunted the maturation and reconstitution of CD14 + myeloid and to some extent CD235a + erythroid cells in the peripheral blood, lymph nodes, spleen and bone marrow of humanized mice. It has been shown that HIV and SIV Nef inhibited differentiation of HSPCs into multiple lineages of hematopoietic cells in macaques, and NEF/PPARγ/STAT5 signaling pathway was found to be involved in this process [40]. In BLT mice, human CD4 + CD8 + thymocytes were found depleted in the presence of LAI Nef, but it was unknown whether LAI Nef exerted a similar effect on myeloid and erythroid cells. Alternately, LAI Nef may also block the development of HSPCs resulting in compromised differentiation of multiple lineage cells. Considering the results obtained in current study and the finding in another study of ours that Nef also blocked the development of T cells, we speculate that the point where Nef blocks hematopoietic cell differentiation is at the level of HSPC differentiation into distinct hematopoietic lineages [41]. In consideration of the PI and Annexin V staining results of LVX-nef transduced HSPCs, we believe that differentiation block and premature death of HSPCs by transduced Nef accounted for the findings in our study.

Damage to the HSPCs in HIV-1 infected patients by Nef probably occurs very early during infection before the start of ART. In those infected individuals on successful ART persistent low level of viral replication was observed and both R5 and X4 tropic HIV-1 genomes have been detected in HPSCs. Based on the decay kinetics of residual viremia in these virally-suppressed patients, stem cells are proposed to serve as a viral reservoir and continue producing viral particles at a low level. Indeed, we could easily detect *nef* expression in LVX-nef transduced, un-sorted HSPCs by western blot but not in HIV-1_NL4-3_ exposed HSPCs (Fig. 1a). Accordingly, *nef* mRNA level was 102 times higher in LVX-nef transduced, un-sorted HSPCs than HIV _NL4-3_ exposed HSPCs (Additional file 1: Fig. S3). Via an ultra-sensitive method p24 antigen could be detected in the culture supernatant of HIV _NL4-3_ exposed HSPCs (5.753 pg/ml), in which CD3 + cells was determined to be less than 0.05% in the HIV _NL4-3_ exposed HSPCs (data not shown). In addition, P24 protein was detected in CD34 + HSPCs by immunofluorescence staining (Additional file 1: Fig. S4). Thus, although the efficiency was low, HSPCs could be infected by X4 tropic HIV-1 in vitro. Besides, recent advances have established that extracellular Nef can accumulate inside uninfected cells through several mechanisms, possibly bypassing infection of HSPCs as the mechanism of hematopoietic abnormalities. With the wide availability of anti-HIV medications to infected individuals worldwide and the effectiveness of these anti-viral drugs, almost every patient receiving ART can achieve very good suppression of viral replication but not eradicating HIV-1 infection. Thus, one of the challenges in the new era of HIV-1 disease management is how to control the co-morbidities such as hematopoietic abnormalities associated with chronic HIV-1 infection.

It is possible that none of the known activities of Nef are associated with inhibition of hematopoiesis as clear associations of Nef functions with in vivo pathogenic effects have not yet been described [42]. Therefore, further studies are needed to understand the mechanism of Nef and the corresponding cellular factors in the pathogenesis of hematopoietic abnormality induced by HIV-1 infection, and our study demonstrates that therapeutics targeting viral protein Nef would be promising in correcting hematopoietic abnormalities in HIV-1 infected individuals.

## Conclusions

Our results demonstrate HIV-1_LAI_ Nef and the Nef from a primary isolate compromised the development of myeloid-erythroid lineage cells. These results add to our understanding HIV pathogenesis and novel therapeutics targeting Nef would be developed in correcting HIV-1 associated hematopoietic abnormalities.

## Methods

### Isolation of human CD34 + cells

Human CD34 + cells were enriched from umbilical cord blood samples and the mothers were tested negative for HIV, HBV, HCV and *Treponema pallidum*. Informed consent was obtained from each mother and the study was approved by the Institutional Ethics Review Board of the 1^st^ Affiliated Hospital of Nanchang University. Cord blood was separated on a Ficoll-Hypaque plus density gradient (GE healthcare) and umbilical cord blood mononuclear cells (UCBMC) were isolated. The isolated UCBMCs were then subjected to magnetic separation to enrich CD34 + cells (Miltenyi Biotec, Cat. No.: 130–046-702).

### Lentivirus preparation

HIV-1_LAI_ (LAI) *nef* (NCBI accession No.: K02013) or the *nef* from a primary isolate was cloned into pLVX-EF1a-IRES-Zsgreen1 (Clontech) via EcoR I and BamH I sites. Lentiviruses were prepared by transfection of pLVX-EF1a-(NEF)-IRES-Zsgreen1, the packaging vector (psPAX2) and the VSVG envelop expressing plasmid into 293 T cells. Three days after transfection viral supernatant was harvested and concentrated by Lenti-X Concentrator (Clontech) according to manufacturer’s instructions. Viral titer was determined by transduction of 293 T cells [43].

### Lentiviral transduction

Human CD34 + cells enriched from cord blood were transduced with LVX-vector or LVX-nef as described previously [44]. In brief, a 96-well non-tissue-culture-treated plate was coated with RetroNectin (Clontech) at 100 μg/mL in PBS for 2 h at room temperature (RT). The wells were then blocked with 2% (w/v) BSA in PBS for 30 min at RT. After aspiration of BSA solution, lentiviruses were added to each well at a total volume of 150 μl. The plate was then centrifuged at 3,700 r.p.m. for 2 h at RT. After centrifugation viral supernatant was aspirated, and 300,000 CD34 + cells were added to each well in 150 μl of culture medium consisting of 100 ng/mL of stem cell factor (R&D), 100 ng/mL of Flt-3 ligand (R&D), 20 ng/mL of IL-6 (R&D), 20 ng/mL of TPO (R&D), 10 ug/mL of heparin (Sigma), 0.5 mmol/L of monothioglycerol (Sigma) and 8 ug/mL of polybrene (Sigma) in Stemspan media (Stemcell technology). The plate was centrifuged again at 1,300 r.p.m. for 10 min at room temperature and then transferred to a 37 °C incubator for viral transduction. Medium containing polybrene was replaced with fresh medium containing the above cytokines 24 h after transduction. Transduction efficiency was checked 72 h after transduction for ZsGreen1 expression and CD34 + ZsGreen1 + cells were sorted out by a flow cytometer (Beckman MoFlo XDP) for downstream applications.

### Western blotting for Nef protein expression

HSPCs transduced with vector or nef-expressing lentiviruses or infected with NL43 were washed twice in ice-cold PBS, pelleted and lysed in RIPA buffer (150 mmol/L NaCl, 1.0% Nonidet P-40, 0.1% sodium dodecyl sulfate, 50 mmol/L Tris–HCl, pH 8.0). Protein concentration was determined using a BCA protein assay kit (Bio-Rad, Hercules, CA). Whole-cell lysates of 70 μg of protein were electrophoretically separated by 12% sodium dodecyl sulfate–polyacrylamide gel electrophoresis and then electrotransferred to the HyBond-P membrane (Millipore). Proteins on the membrane were detected with an anti-mouse Nef primary antibody and an appropriate peroxidase-labeled secondary antibody followed by the ECL chemiluminescence reagents (Amersham). Both primary and secondary antibodies were from Abcam. β-actin was used as a loading control.

### Colony forming assay (CFA)

The assay was set up according to manufacturer’s instructions (Stemcell technology). In brief, CD34 + cells were diluted with IMDM + 2% FBS to 2 × 10^4^/mL. 0.3 mL of diluted cells was added to 3 mL of MethoCult™ media (H4034, Stemcell technology) for a duplicate assay. Cells and media were then vortexed vigorously to thoroughly mix the content, and stood for at least 5 min to allow the bubbles to rise to the top. 1.1 mL of cell-MethoCult™ medium mixture was dispensed into a 35 mm dish. Two 35 mm dishes were placed together with an uncovered 35 mm dish with sterile water inside into a 100 mm dish, and incubated at 37 °C, 5% CO2 for 14—16 days. After incubation colonies were scored based on morphology with an Olympus microscope and then harvested for downstream analysis. For cytospin and Giemsa staining, the same class of colonies were picked individually and then combined. For flow cytometry analysis, colonies were all pulled together from the whole plate.

### Reverse transcription and qPCR for nef mRNA expression

Cells recovered from the 35 mm plate of CFA were subjected to RT-qPCR for *nef* expression. Total RNA was extracted by Trizol reagent (Thermal Scientific), and reverse-transcribed to single stranded cDNA with random oligo dT and hexamer primers after erasion of genomic DNA (Takara). The synthesized cDNA was then subjected to qPCR amplification (Takara) with LAI *nef* specific primers and probe (forward: 5’- AGCTACCAATGCTGCTTGTG-3’; Reverse: 5’-GGTACCTGAGGTGTGACTGG-3’; 6-FAM- ACCCACCTCCTCCTCCTCTTGTGC-TAMRA). GAPDH was used as an internal control (forward: 5’-ACCCAGAAGACTGTGGATGG-3’; Reverse: 5’-TCAGCTCAGGGATGACCTTG-3’; 6-FAM-CCCACAGCCTTGGCAGCGCC-TAMRA). The cycling condition was 95℃ 30 s followed by 40 cycles of 95℃ 5 s and 60℃ 30 s. Data were collected in a CFX96 real-time PCR detection system and analyzed with the Bio-Rad CFX manager software (Bio-Rad).

### Cytospin and Giemsa staining

Cells of harvested colonies from the colony forming assay were transferred to a slide using a cytospin centrifuge at 500 rpm for 5 min at room temperature. Slides were then stained with Giemsa staining with a standard protocol. Photomicrographs were taken with an Olympus camera with a 100 × oil objective.

### Immunofluorescence staining (IF) and confocal microscopy imaging

CD34 + HSPCs either transduced with nef-expressing lentiviruses or infected with NL43 were transferred to a slide by cytospin. IF was performed with a standard protocol. For nef (zsgreen1) expression slides were stained with a rabbit anti-human CD34 antibody (Abcam) and an Alexfluor 555 conjugated second antibody (Thermal fisher). For intracellular P24 expression slides were stained with a mouse anti-P24 antibody (Immunoway) and an Alexfluor 488 conjugated second antibody (Thermal fisher) after surface staining for CD34. All the slides were finally counter-stained with DAPI and photomicrographs were taken with an Olympus confocal microscopy with a 100 × oil objective.

### Flow cytometry analyses of erythroid and myeloid lineage cells of recovered colonies

Cells pulled from the whole 35 mm plate of CFA assay were subjected to flow analysis with mouse anti-human CD45 PE-Cy7 and CD235a antibodies for erythroid and CD45 PE-Cy7, CD33 APC and CD14 PE antibodies (BD Pharmigen) for myeloid differentiation analysis. Data were analyzed with Flowjo version 10.

### Mice

NCG mice [45, 46]were purchased from the Model Animal Research Center of Nanjing University. This model was created by sequential CRISPR/Cas 9 editing of the *Prkdc* and *Il2rg* loci in the NOD/Nju mouse. The NOD/Nju mouse carries a mutation in the *Sirpa* gene that allows for engrafting of foreign hematopoietic stem cells. The *Prkdc* knockout generates a SCID-like phenotype lacking proper T cell and B cell formation. The knockout of the *Il2rg* gene further exacerbates the SCID-like phenotype while additionally resulting in a decrease of NK cell production. Mice were housed in a specific pathogen-free facility at Jiangxi Academy of Medical Sciences according to Institutional Animal Care and Research Committee-approved protocols. Mice were maintained in Individual Ventilated Cages and fed with sterile food and sterile, chlorinated water.

### Irradiation and transplantation

6–8 weeks’ old NCG mice were irradiated (250 Rads from an X-ray radiation source) and then transplanted intravenously within 6 h of irradiation with 3–5 × 10^5^ of CD34 + cells transduced either with control vector or *nef-*expressing lentiviruses. Engraftment and reconstitution of human cells were assessed every 4 weeks after transplantation for 12 weeks by monitoring the percentage of human CD45 + cells in peripheral blood. Myeloid and erythroid human cell subsets were also phenotypically characterized every 4 weeks by flow cytometry. At 12 weeks after transplantation, mice were sacrificed and tissues were harvested for flow cytometry and immunohistochemical analysis.

### Human reconstitution analysis by flow cytometry

Mononuclear cells were isolated from peripheral blood, bone marrow, spleen, and lymph nodes of the reconstituted mice. The percentage of human leukocytes (CD45 +), erythroid and myeloid lineage cells were determined by flow cytometry using antibodies to human specific markers. Flow cytometry data were collected and analyzed with flowjo version 10.The gating strategy was live > CD45-CD235a + for erythroid and live > Zsgreen +  > CD45 + CD14 + CD33 + for myeloid cells, respectively.

### Haematoxylin and eosin (H&E) and immunohistochemical analysis

Mouse lymph nodes and spleens were collected and placed in fresh phosphate-buffered 4% paraformaldehyde for 24 h, and then embedded in paraffin. Hematoxylin and eosin (H&E) and immunohistochemistry (IHC) staining for CD45 + and CD14 + cells in these tissues was conducted to analyze human myeloid cell reconstitution. IHC staining for Nef in spleens was conducted to confirm the expression of Nef in the humanized mice transplanted with *nef-*expressing HSPCs. Photomicrographs were taken with an Olympus camera with 10 × and 40 × optical objectives.

### Data acquisition and statistical analysis

Immunohistochemistry positive cell numbers were obtained using stereological best practices. Briefly, positively stained cells were counted on a systemically random sample of three sections by three independent individuals using a view field of 100 × 100 μm (a two-dimensional grid) with NIH Image J software (http://rsb.info.nih.gov.ij). The cell counting was performed on at least three animals of each group, and three different view fields were chosen randomly within each section of the tissue. Average values of cell counts were calculated from the pooled data. To ensure the objectivity, all sections were blinded for their number and treatments, and the labels were opened only after the data acquisition was complete. All values were expressed as mean ± SEM. Comparisons between groups were made using two-tailed Student’s *t*-test in Prism version 6 (GraphPad Software, Inc.). A *p* value of < 0.05 was considered statistically significant (*), *p* < 0.01 highly significant (**), and *p* < 0.001 extremely significant (***).

## Supplementary Information

**Additional file 1.** Supplementary figures.

## Data Availability

All data generated or analyzed during this study are included in this published article (and its supplementary information files), and are available from the corresponding author on reasonable request.

## Abbreviations

HIV-1: Human immunodeficiency virus-1
Nef: Negative factor
HSPCs: Hematopoietic stem/progenitor cells
AIDS: Acquired immunodeficiency syndrome
ART: Antiretroviral therapy
CFA: Colony forming assay
BLT: Bone/liver/thymus
M-CSF: Macrophage colony-stimulating factor
PI: Propidium iodide
IF: Immunofluorescence staining
