# Supplementary material for: HIV-1LAI Nef blocks the development of hematopoietic stem/progenitor cells into myeloid-erythroid lineage cells
Source: Biol Direct. 2021 Dec 20;16:27. doi: 10.1186/s13062-021-00317-3 (PMC8686389; doi:10.1186/s13062-021-00317-3)
Supplement: Supplementary file 1 — Additional file 1. Supplementary figures. [file 13062_2021_317_MOESM1_ESM.doc]

Supplementary figures

**Fig. 1. Relative *nef* mRNA expression in the colonies developed from LVX-vector or LVX-*nef* transduced CD34+ cells.** LVX-vector or LVX-*nef* transduced CD34+ cells were cultured in methylcellulose-based media for lineage development. After 14 days colonies were harvested, and total RNA was extracted. The synthesized cDNA was then subjected to qPCR for *nef* expression with *nef* specific primers and probe. The relative expression of *nef* mRNA was calculated by ∆∆Ct method with *GAPDH* as the normalization control. A representative experiment was presented in the figure.

**Fig.2. DNA sequence alignment of *nef* from LAI and the HIV-1 primary isolate tested in our study.**


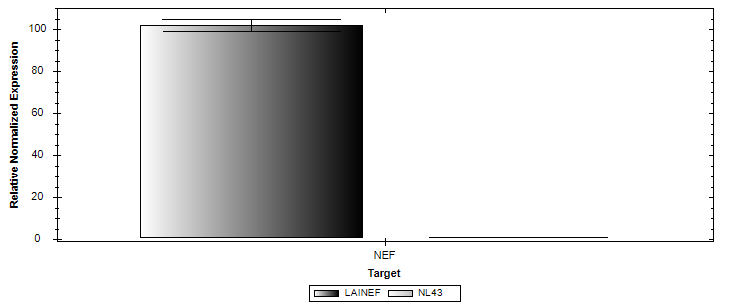


**Fig. 3. Relative *nef* mRNA expression in the HSPCs either transduced with LVX-nef or exposed to HIV-1_NL43._** Total RNA from the HSPCs either transduced with LVX-Nef or exposed to HIV-1_NL43_ was extracted. The synthesized cDNA was then subjected to qPCR for *nef* expression with *nef* specific primers and probe (n=3). The relative expression of *nef* mRNA was calculated by ∆∆Ct method with *GAPDH* as the normalization control.

**Fig.4. HIV-1 P24 expression in CD34+ HSPCs exposed to HIV-1_NL43._** CD34+ HSPCs infected with NL43 were transferred to a slide by cytospin. Cells were stained with a mouse anti-P24 antibody and an Alexfluor 488 conjugated second antibody after surface staining for CD34. Cells were finally counter-stained with DAPI and photomicrographs were taken with an Olympus confocal microscopy with a 100x oil objective.
